# Supplementary material for: MetaRibo-Seq measures translation in microbiomes
Source: Nat Commun. 2020 Jun 29;11:3268. doi: 10.1038/s41467-020-17081-z (PMC7324362; doi:10.1038/s41467-020-17081-z)
Supplement: Supplementary file 10 — Supplementary Data 7 [file 41467_2020_17081_MOESM10_ESM.zip › File2/Confidence_High_Taxonomy/84105_out.krona.html]

Javascript must be enabled to view this page.

members
magnitude
magnitudeUnassigned
count
unassigned
taxon
rank

84105\_out

14

14
2
superkingdom

14
976
phylum

14
200643
class

171549
14
order

family
171552
14

genus
838
14

species

SRS012989\_contig\_number\_3943SRS013638\_contig\_number\_2440SRS017375\_contig\_number\_12SRS023914\_contig\_number\_2592SRS046502\_contig\_number\_12723SRS050752\_contig\_number\_2721SRS074586\_contig\_number\_2527SRS077194\_contig\_number\_10540SRS077641\_contig\_number\_13SRS077849\_contig\_number\_13600SRS077991\_contig\_number\_12SRS1041134\_contig\_number\_1048SRS147614\_contig\_number\_1993SRS148253\_contig\_number\_8627
165179
14
